# A detailed analysis of anatomical plausibility of crossed and uncrossed streamline rendition of the dentato-rubro-thalamic tract (DRT(T)) in a commercial stereotactic planning system

**DOI:** 10.1007/s00701-021-04890-4

**Published:** 2021-06-28

**Authors:** Volker A. Coenen, Bastian E. Sajonz, Peter C. Reinacher, Christoph P. Kaller, Horst Urbach, M. Reisert

**Affiliations:** 1 Department of Stereotactic and Functional Neurosurgery, Medical Center of Freiburg University, Breisacher Strasse 64, 79106 Freiburg i.Br, Germany; 2 Medical Faculty of Freiburg University, Freiburg, Germany; 3 Center for Deep Brain Stimulation, Medical Center of Freiburg University, Freiburg, Germany; 4 Fraunhofer Institute for Laser Technology, Aachen, Germany; 5 Department of Neuroradiology, Freiburg University Medical Center, Freiburg, Germany; 6 Department of Radiology - Medical Physics, Freiburg University, Freiburg, Germany

**Keywords:** Brain, Diffusion tensor imaging, Tractography, Stereotaxy, Deep brain stimulation, DBS, DRT

## Abstract

**Background:**

An increasing number of neurosurgeons use display of the dentato-rubro-thalamic tract (DRT) based on diffusion weighted imaging (dMRI) as basis for their routine planning of stimulation or lesioning approaches in stereotactic tremor surgery. An evaluation of the anatomical validity of the display of the DRT with respect to modern stereotactic planning systems and across different tracking environments has not been performed.

**Methods:**

Distinct dMRI and anatomical magnetic resonance imaging (MRI) data of high and low quality from 9 subjects were used. Six subjects had repeated MRI scans and therefore entered the analysis twice. Standardized DICOM structure templates for volume of interest definition were applied in native space for all investigations. For tracking BrainLab Elements (BrainLab, Munich, Germany), two tensor deterministic tracking (FT2), MRtrix IFOD2 (https://www.mrtrix.org), and a global tracking (GT) approach were used to compare the display of the uncrossed (DRTu) and crossed (DRTx) fiber structure after transformation into MNI space. The resulting streamlines were investigated for congruence, reproducibility, anatomical validity, and penetration of anatomical way point structures.

**Results:**

In general, the DRTu can be depicted with good quality (as judged by waypoints). FT2 (surgical) and GT (neuroscientific) show high congruence. While GT shows partly reproducible results for DRTx, the crossed pathway cannot be reliably reconstructed with the other (iFOD2 and FT2) algorithms.

**Conclusion:**

Since a direct anatomical comparison is difficult in the individual subjects, we chose a comparison with two research tracking environments as the best possible “ground truth.” FT2 is useful especially because of its manual editing possibilities of cutting erroneous fibers on the single subject level. An uncertainty of 2 mm as mean displacement of DRTu is expectable and should be respected when using this approach for surgical planning. Tractographic renditions of the DRTx on the single subject level seem to be still illusive.

## Introduction

**Fig. 1:**
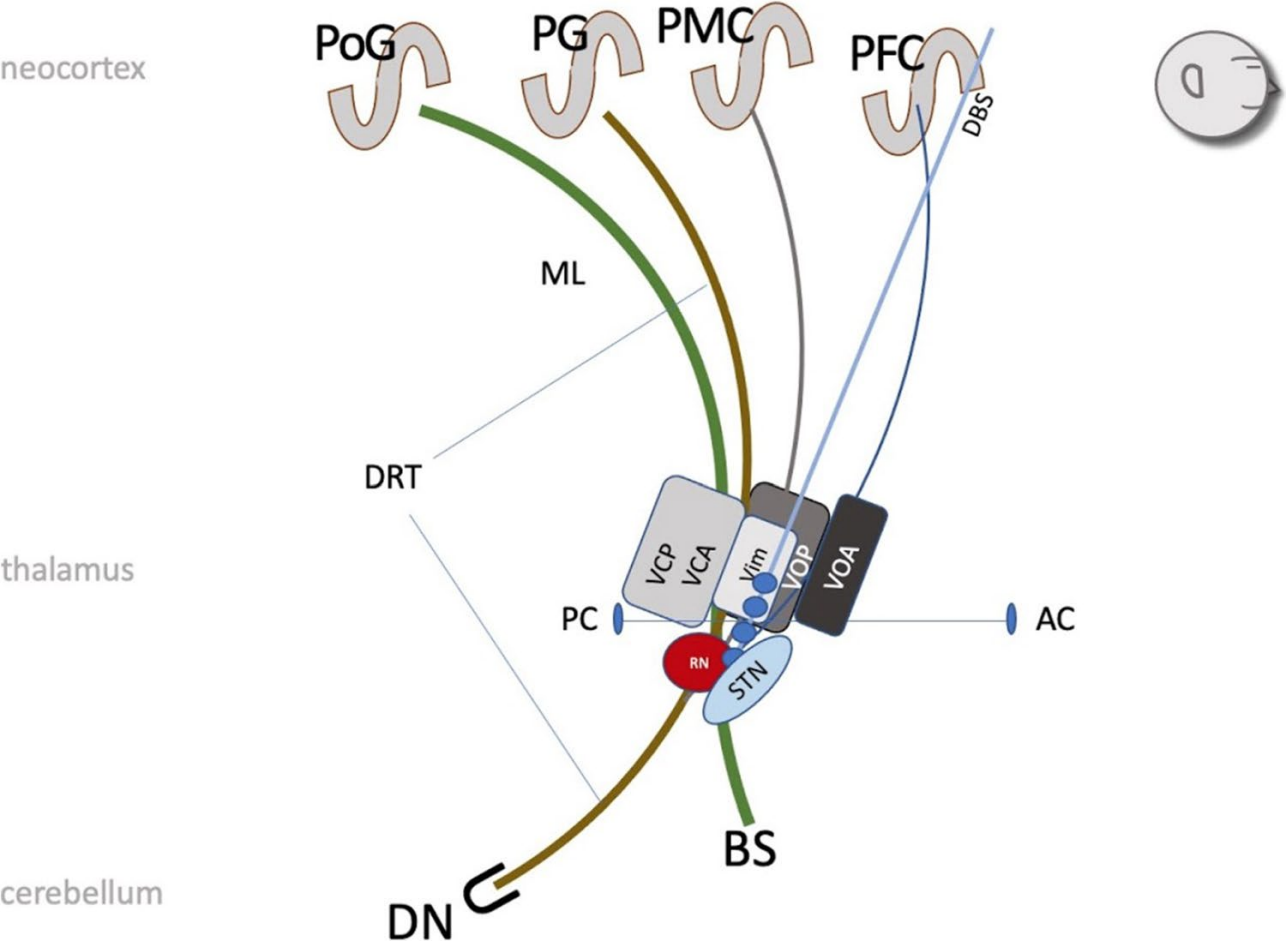
Principle and schematic anatomical course of the DRT (brown) between dentate nucleus (DN) and (contralateral) precentral gyrus (PG)* and adjacent structures (sagittal view). Legend: BS, brain stem; AC, anterior commissure; PC, posterior commissure; DBS, deep brain stimulation electrode; PFC, prefrontal cortex; PMC, premotor cortex; PoG, postcentral gyrus; ML, medial lemniscus; RN, red nucleus; STN, subthalamic nucleus; VOA, ventral oral anterior nucleus (ot; of thalamus); VOP; ventral oral posterior nucleus (ot); Vim, ventral intermediate nucleus (ot); VCA, ventral caudal anterior nucleus (ot); VCP, ventral caudal posterior nucleus (ot). * of note, DRTx might have slightly different projection fields

Stereotactic surgery for tremor of various origins has typically targeted the ventral intermediate nucleus of the thalamus (Vim) [7, 71]. The relationship between mere thalamic DBS electrode positions and outcome has been addressed [46]. There is a growing interest in augmenting classical stereotactic surgical procedure planning on the single subject level with refined imaging strategies in order to improve safety and efficacy of deep brain stimulation (DBS) in its various neuropsychiatric indications [13, 18, 24, 44, 54, 57, 59]. There is evidence that the effective target of tremor surgery might be one distinct part of the tremor network which can readily be depicted with non-invasive diffusion weighted imaging (dMRI) technology [15]. This target is the cerebello-thalamo-cortical pathway—by some authors also called the dentato-rubro-thalamic tract (DRT) (Fig. 1)—which in fact penetrates all classical stereotactic targets for tremor surgery [14, 15], namely the ventral intermediate nucleus of the thalamus [6, 7] and the posterior subthalamic region (pSTR) [33] including the caudal zona incerta (cZI) [3, 48, 49]. In recent years, there have been group level approaches which retrospectively analyze sweet spots for tremor reduction and aim at defining networks relevant for effective tremor DBS [1, 14, 32, 39, 51, 55]. These studies have confirmed the role of the DRT as a target for tremor DBS.

Although evidence for the DRT as the main tremor reducing structure is convincing [3, 17, 21, 33] on the single subject level and for stereotactic planning procedures, some questions remain unanswered concerning the anatomical plausibility, the definition of the DRT’s thalamic penetration level, and the congruence and reproducibility of DRT rendition with respect to the streamline display in modern commercial stereotactic planning systems and other tracking environments.

Typically, the streamline selection process on a stereotactic planning station is a manual, iterative process and prone to subjective bias. In this contribution, we therefore compare the deterministic DRT streamline reconstruction in a modern stereotactic planning environment (Elements®, Brainlab, Munich, Germany) with two fully automated tracking approaches used for neuroscientific research. More specifically, we used (1) a probabilistic approach, i.e., a common MRtrix pipeline (www.mrtrix.org) using constrained spherical deconvolution with iFOD2 probabilistic tractography, and (2) a global approach, i.e., global tractography based on a Gibbs sampler [52]. Our goal was to evaluate the quality of anatomical DRT streamline rendition under distinct circumstances (different quality imaging sequences) and to quantify and compare the results with a special focus on the quality of the streamline rendition in a direct surgical planning setting.

## Methods

### Data

In total, 9 subjects were included resulting in 15 diffusion MRI (dMRI) datasets as six subjects had been scanned twice. Different MRI scanners and sequences were used in order to evaluate the performance of the individual tracking environments under conditions of high (PRISMA) or low (TRIO) imaging quality.

#### TRIO

Three young normal subjects (24F, 25F, 24 M) were scanned on a Siemens TIM TRIO using a 1-shell protocol with b-value 1000 and 60 directions per shell, at an isotropic resolution of 2 mm, 6/8 partial Fourier, TR = 10,900 ms, and TE = 107 ms. Additionally, a T1-weighted structural dataset was acquired, resolution 1 mm isotropic. This protocol takes about 10 min of measurement time. For distortion correction, the PSF-mapping technique was used [72]. Each subject was scanned twice (in two different sessions) to investigate the robustness and reliability of dMRI measures.

#### PRISMA (CLIN)

Three elderly subjects (78F, 70F, 72 M) were scanned on a Siemens 3 T TIM PRISMA using an SE EPI sequence with a TE = 88 ms and TR = 2800 ms, bandwidth 1780 Hz, flip-angle 90, GRAPPA factor 2, SMS factor 3 with 17 non-diffusion weighted images, 2*58 images with b-factor b = 1000 and 2000s/mm^2^; with an in-plane voxel size of 1.5 mm, 1.5 mm, and a slice thickness of 3 mm. Overall, this EPI protocol takes about 7 min of measurement time. Additional T1-weighted images (TR = 2500 ms, TE = 2.8 ms, 1 mm isotropic) and T2-weighted images (TR = 2500 ms, T1 = 231 ms, 1 mm isotropic) were acquired.

#### PRISMA (CTR)

Three elderly normal subjects (76F, 63F, 56 M) were scanned on a Siemens 3 T TIM PRISMA using two different SE EPI sequences within one session: one with parameters identical to the PRISMA CLIN dataset and a second where the second shell at b = 2000s/mm^2^ is omitted and an isotropic resolution of 1.7 mm is used with an TE = 69 ms and TR = 4100 ms (duration 6 min).

### Preprocessing and anatomical segmentation

In the following, we compare Brainlab’s Elements deterministic 2-tensor deflection streamline tracking approach (FT2) with global tractography (GT) and probabilistic tractography (MRtrix iFOD2). All approaches used Brainlab’s Elements anatomical segmentation as a basis for DRT selection utilizing segmentation masks of the nucleus ruber (RN), the precentral gyrus (PG), and the superior cerebellar peduncle (SCP). Elements Image Fusion (Release 4.0, Brainlab AG, Munich, Germany) was used to rigidly co-register anatomical MRI and DTI data. Automatic segmentation of the RN, SCP, and PG as used for tractography purposes was applied by Elements Segmentation Basal Ganglia (Release 5.0, Brainlab AG, Munich, Germany) as part of Brainlab Elements. This approach is based on a synthetic tissue model which consists of multiple tissue-classes and is flexibly adapted to an individual patient’s anatomy and imaging data, e.g., by performing abnormality and sequence detection (patent US9639938B2). The procedure allows to simulate multiple individualized and (sub-)modality-specific atlases mimicking respective (grey-value) image contrasts visible in the various images (e.g., T1-, T2-, SWI-weighted MRI), and thereby to consider multiple scans with different image information at the same time during the automatic segmentation process. Subsequently, iterative atlas-to-scan registration is performed using nonlinear, elastic mapping of each atlas onto each scan, followed by cranial anatomy segmentation applying individual structure-specific weighting of each atlas-to-scan registration to calculate a final registration field [50].

While the masks which are used in the following for streamline selection are identical for all considered pipelines, the preprocessing of the dMRI data was partly different as described in the following:

#### Preprocessing FT2 elements

To remove noise artifacts outside soft tissue anatomies, a 3D mask was obtained from the baseline “B0” scan and applied to the dMRI data. For eddy current and distortion correction, Brainlab uses an in-house correction, which is based on spatial consistency within the dMRI data. Furthermore, motion correction and B-matrix rotation are applied by affine transformation to compensate for bulk motion and to align anatomies along the anatomical sagittal plane. Finally, a denoising step is applied, which is based on principal component analysis (PCA) to improve the signal-to-noise ratio (SNR) of the dMRI series by considering the local-kernel-weighted and multi-directional signal information at the same time [35].

#### Preprocessing GT and Mrtrix

For the TRIO set, the distortion correction was already applied at scanner level using [72]. For both PRISMA datasets, one phase-encoding flipped b = 0 image was acquired and used for distortion correction using FSL’s topup [4]. The diffusion-weighted images were first denoised by a post-processing technique which uses random matrix theory [31, 67] and is very close to the approach of Brainlab’s Elements [35]. This was followed by Gibbs artifact removal based on local sub-voxel shift [31]. Afterwards, images were corrected for EPI distortions by FSL’s topup.

#### Fiber tracking

The focus of this study was the comparison of manually created DRT tracking results (with a modern, commercial stereotactic planning system, i.e., Brainlab Elements) with the fully automated selection from two tracking approaches used in neuroscientific research, namely (1) a simple MRtrix pipeline using constrained spherical deconvolution [65] and the common iFOD2 tracking [66] and (2) global tractography (GT) based on a Gibbs sampler [52].

#### Elements

Release 2.0 (Brainlab AG, Munich, Germany) was used to perform semi-automatic tractography of DRTu and DRTx by means of an automated built-in DTI post-processing routine for atlas-segmentation-based customizable template application. The tracking procedure is very close to the deterministic FACT algorithm [42] together with a simple post-processing [69]. Definition of “include-regions,” i.e., seed volumes (see below), as well as stopping criteria for deterministic fiber tracking are determined (FA level for cut off, 0.15–0.2; minimal length 50–80 mm; maximal angulation 20°).

**Fig. 2:**
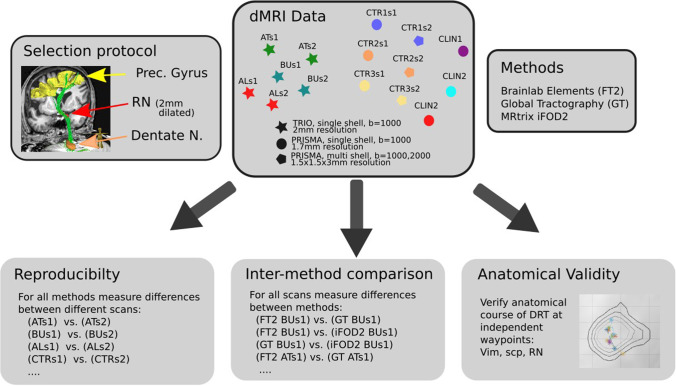
Data evaluation strategy. Three methods are compared with respect to their robustness, plausibility, and agreement of the DRT streamline course. Fifteen diffusion scans from two types of scanners (Siemens TIM TRIO/PRISMA) are analyzed. Six scans from TRIO from three subjects (AT, BU, SL), where each subject was scanned twice (s1/s2). Similarly from PRISMA, but three subjects (CLIN1, CLIN2, CLIN3) only scanned once

The manual or semi-automated selection protocol in the commercial elements environment utilized a selection of starting (dentate nucleus, DN) and end volumes (precentral gyrus, PG; either ipsi- for DRTu or contralateral for DRTx). As a waypoint volume, the red nucleus (RN, either ipsi- for DRTu or contralateral for DRTx) was used. Since the DRTu/x only passes/touches the RN in its posterior lateral part (Fig. 1), the RN outline was increased by 2 mm. The resulting streamlines were then checked for anatomical plausibility. Because the RN volume was increased circumferentially, parts of the structures in the Forel’s fields H [27] were jointly tracked (mainly the superolateral medial forebrain bundle, slMFB, and ansa lenticularis, AL) [5, 16, 19, 61]. These erroneous fibers of the raw results were then manually eliminated based on anatomical assessment of the resultant structure leading to clean results. Of note, the Vim (ventral intermediate nucleus of thalamus) was not included as way-point volume in the selection procedure (Fig. 2).

The automatic protocol was designed similarly, but naturally refrained from any manual correction: Streamlines which visit the PG, a 2-mm dilated RN, and the contralateral DN were selected as DRT streamlines.

#### MRtrix iFOD2

We used one of the most common tractography pipelines based on MRtrix (www.mrtrix.org) using constrained spherical deconvolution (CSD). For single shell data, standard CSD following [65] was used. Multiple shell data was processed by its multi-tissue pendant [30]. To estimate fiber response function, we relied on [22] for the multi tissue. To reconstruct the DRT, we seeded in whole-brain white matter one million tracts using the default iFOD2 tracking parameters [66]. After reconstruction, we applied the selection protocol described above. A mask for whole-brain white matter (WM mask) was estimated using CAT12 (see below) at a threshold of 0.2.

#### Global tracking

As opposed to a local walker-based tractography, global fiber tracking has a more concrete objective: It creates a fiber configuration that fits to the acquired diffusion-weighted MRI data in the best possible way [26, 52, 62]. The optimization process is similar to a polymerization process; streamlines are initially short and fuzzy, whereas during optimization, the connections proliferate and fibers become increasingly congruent with the data. The algorithm is based on so-called simulated annealing. We followed the publicly available method proposed in [52] (http://www.uniklinik-freiburg.de/mr-en/research_groups/diffperf/fibertools.html).

In this study, we applied the “dense” parameter set and used the same WM mask as for the MRtrix pipeline. To increase stability in terms of higher retest reproducibility, we followed an approach proposed by [62]. Using an accumulation factor of 10, a single whole-brain tractogram comprised approximately 0.7 million streamlines.

#### Group analysis

We analyzed three different aspects: the reproducibility of the methods, the congruence (difference/agreement) between the methods, and the anatomical validity. All evaluations were performed in MNI space. The diffeomorphic mapping between subject and template space was established using CAT12, http://dbm.neuro.uni-jena.de/cat12/CAT12-Manual.pdf. To get tracking results into MNI space, streamline densities were rendered in native space and transformed to MNI space by the CAT12 warp. To quantify the similarity between two tracking results, the streamline densities were subdivided along the MNI z-coordinate from level z =  − 27 to z = 16, which included the regions important in the context of DRT-driven stereotactic targeting. We considered two options to measure the congruence: (1) The Euclidean distance of the center of gravity (COG) by computing the 2D center of gravity for each xy-slice with respect to the fiber density as mass function. (2) The dice coefficient between pairs of binary masks by thresholding the fiber density maps at 2 streamlines per voxel.

## Results

**Fig. 3:**
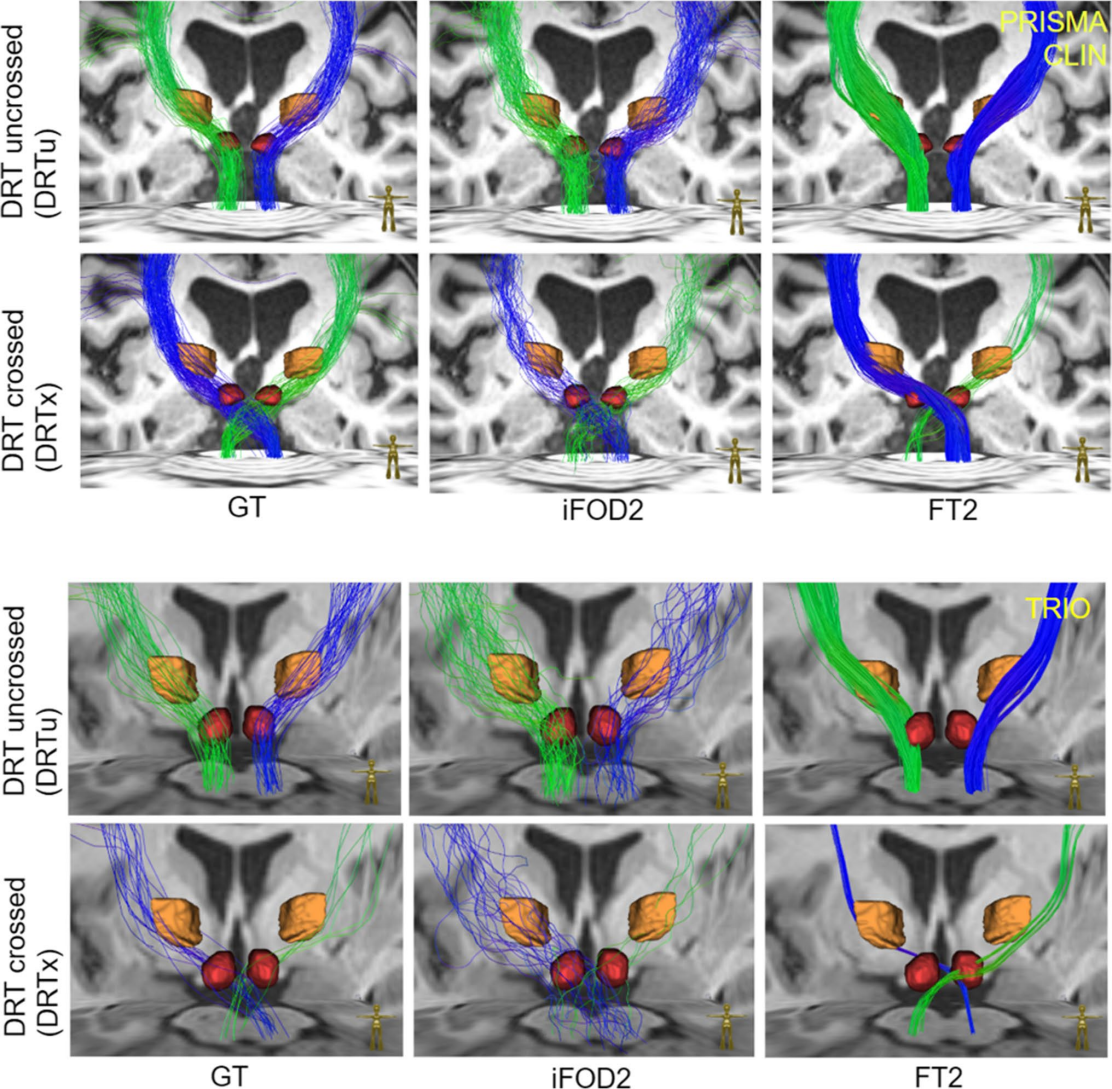
Two representative examples of uncrossed and crossed DRT: on the upper panel, a “good” example from the PRISMA dataset, where GT and iFOD2 successfully reconstruct both DRTs, while Brainlab’s FT2 has problems with one of the crossing tracts. On the lower panel, an example from a qualitatively inferior TRIO dataset is shown, where the uncrossed tract is typically well reconstructed, but it is difficult to get good and reliable results for the crossed one

In Fig. 3, we show two representative examples of DRT reconstructions for the three different methods. Typical results for PRISMA data agree well between the methods for the DRTu. However, problems appear for DRTx, in particular for FT2, where the reconstruction fails quite often; i.e., only a very low number of streamlines is found or no streamlines at all. For TRIO data, also GT and iFOD2 have serious problems to reconstruct DRTx, while DRTu remains mostly valid. These results are not astonishing; due to the better gradient coils (80mT/m), images from PRISMA have a substantially higher quality and better resolution than the data from TRIO, which uses only 40mT/m gradients. In particular, the low echo times achievable with PRISMA lead to less susceptibility induced distortions and better SNR.

### Inter-method comparison on congruence

**Fig. 4:**
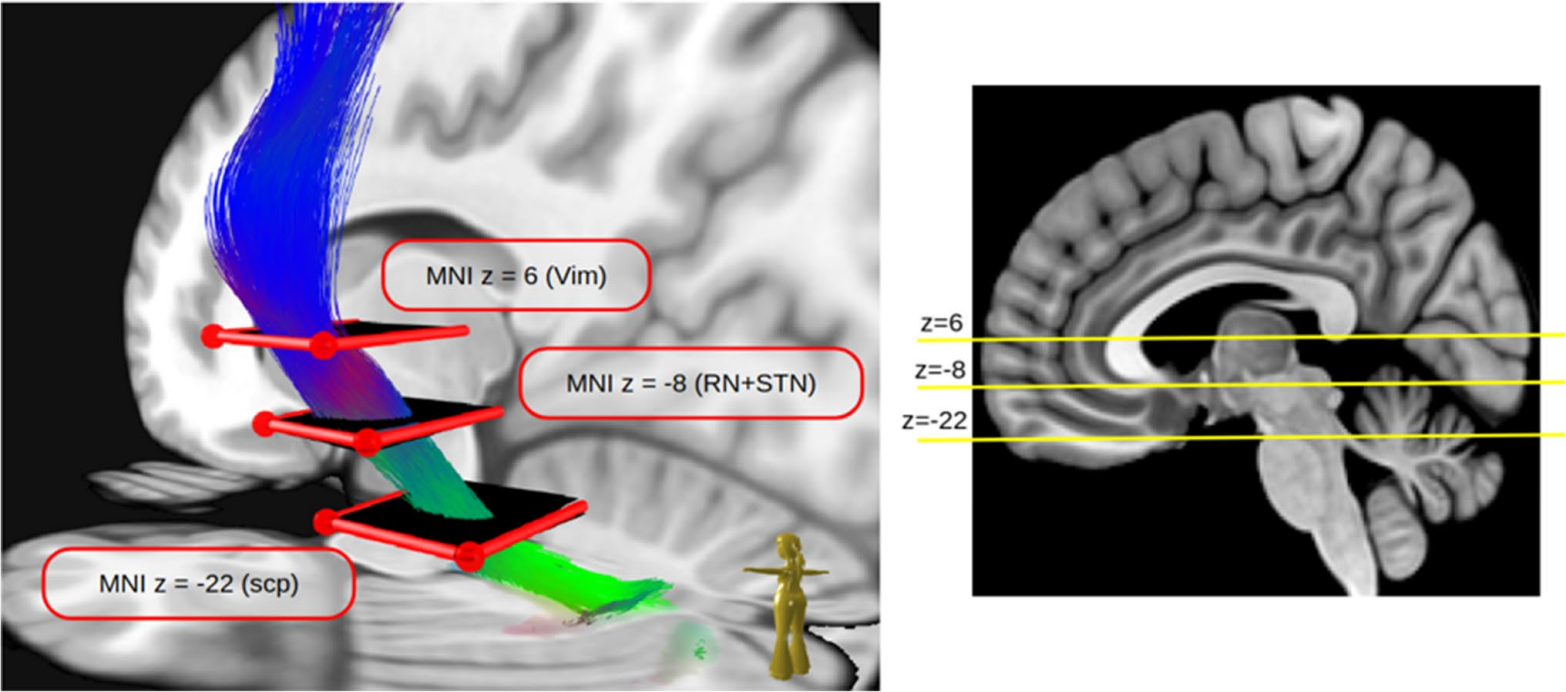
The transversal levels in MNI space we considered for detailed evaluation. The first is at level z = 6 of the ventral intermediate nucleus (Vim), the second at z =  − 8 at the nucleus ruber (RN) and subthalamic nucleus (STN), and finally at z =  − 22 where the superior cerebellar peduncle (SCP) can be visualized best

**Fig. 5:**
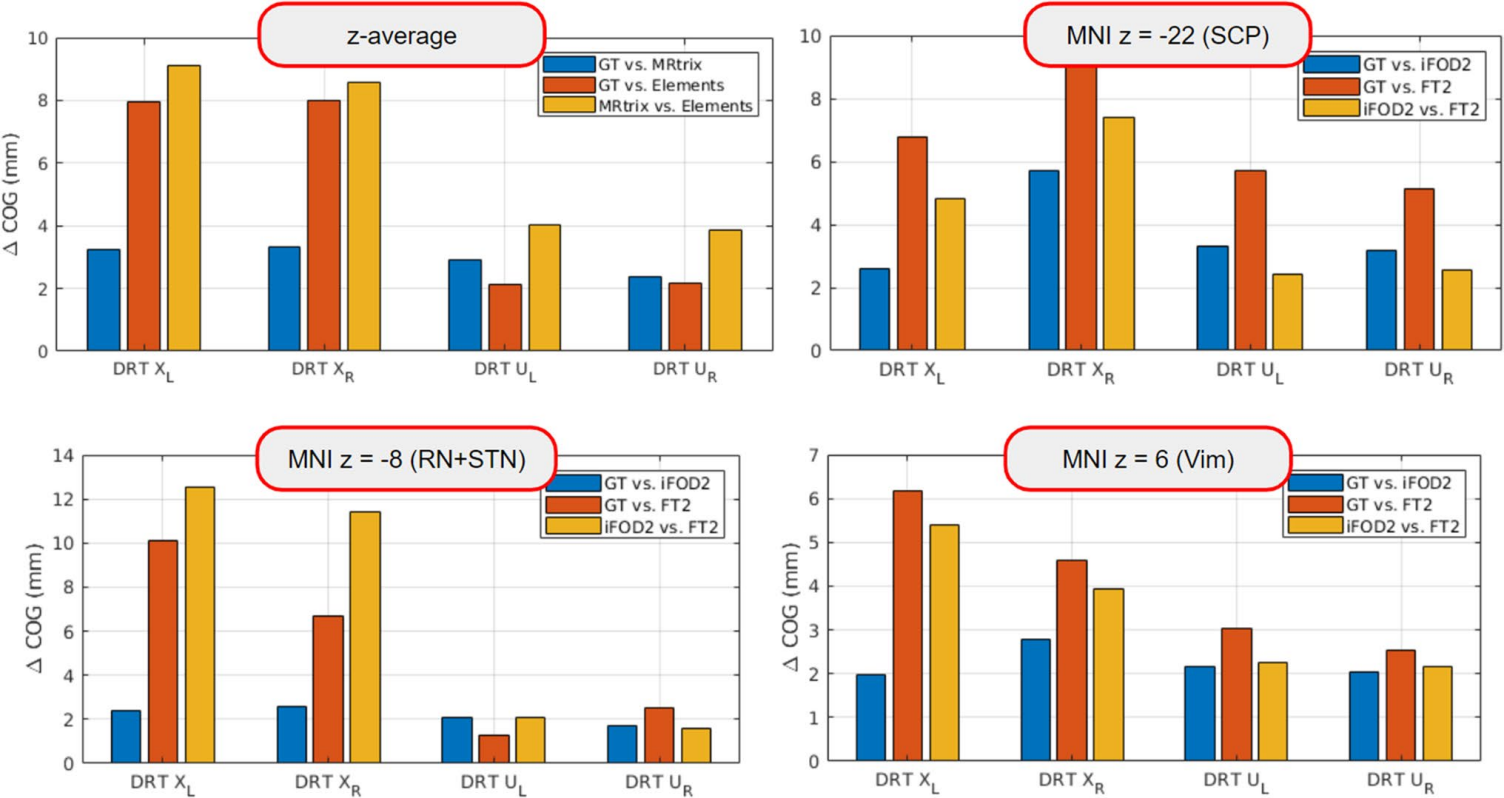
Inter-method comparison by COG differences at the three different levels together with the z-mean from z =  − 27 to z = 16. All methods agree well for the uncrossed DRT, but have differences for the crossed one. While GT and MRtrix’s iFOD2 still show some agreement, Brainlab’s FT2 shows substantial differences compared to GT and iFOD2. Detailed results including dice coefficients can be found in Fig. 8

Dice coefficients and COG differences were computed between all different method pairings to quantitatively compare the DRT renditions of the three tracking approaches. Figure 8 shows detailed z-resolved results for the crossed and unilateral DRT for all pairings. We show the median of the COG deviations along the z-coordinate (Fig. 4) together with three distinct z-levels in Fig. 5 (z =  − 22, superior cerebellar peduncle, scp, z = 6 ventral intermediate nucleus Vim, z =  − 8 nucleus ruber RN, MNI coordinates) as bar plots. The results confirm the qualitative findings from above. The largest deviations happen between MRtrix’s iFOD2 and Brainlab’s FT2, while GT lies in between the two.

### Reproducibility

**Fig. 6:**
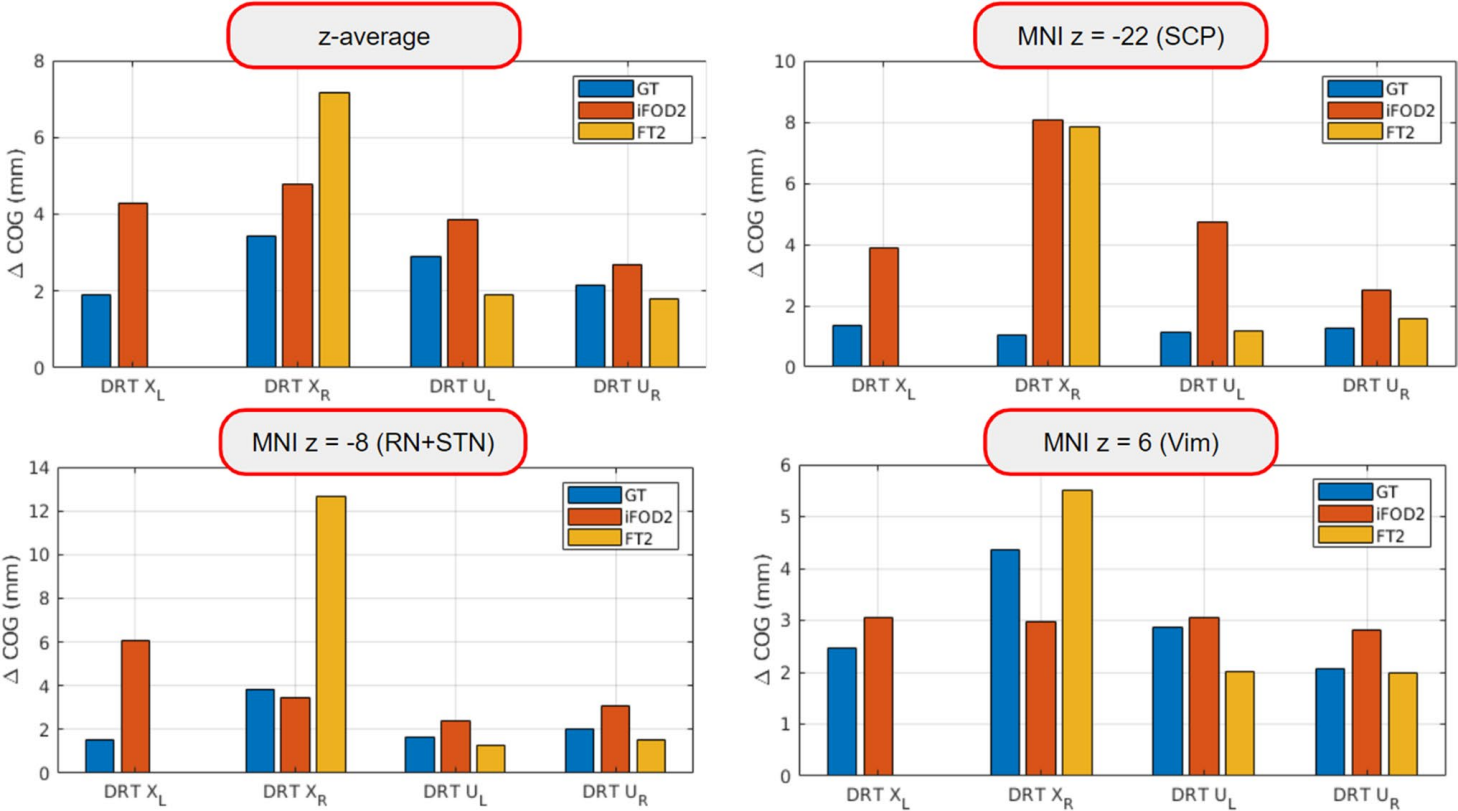
Repeatability measured by COG differences at the three different levels together with the z-mean from z =  − 27 to z = 16. All methods show robust results for the uncrossed DRT (DRTu, accuracy in the range of 2 to 4 mm), but have problems with the crossed one (DRTx)

We also used the above similarity measures to assess repeatability by comparing session 1 with session 2 of the TRIO dataset, and the anisotropic EPI with the isotropic EPI measurement from the PRISMA CTR dataset. In Fig. 9, the COG differences and dice coefficients are shown for the crossed and unilateral DRT for the three different methods. Similarly to the inter-method comparison, Fig. 6 shows the mean along the z-coordinate together with the three z-levels from above. The repeatability appears to be especially insufficient for DRTx when compared in the Vim (MNI z = 6) and in the RN + STN (MNI z =  − 8) levels. The largest error for DRTx repetition is actually seen for FT2. These results are especially concerning since in most surgical planning approaches, the DRT-renditions are used to identify thalamic Vim and the subthalamic region targets (STR).

### Anatomical validity

**Fig. 7:**
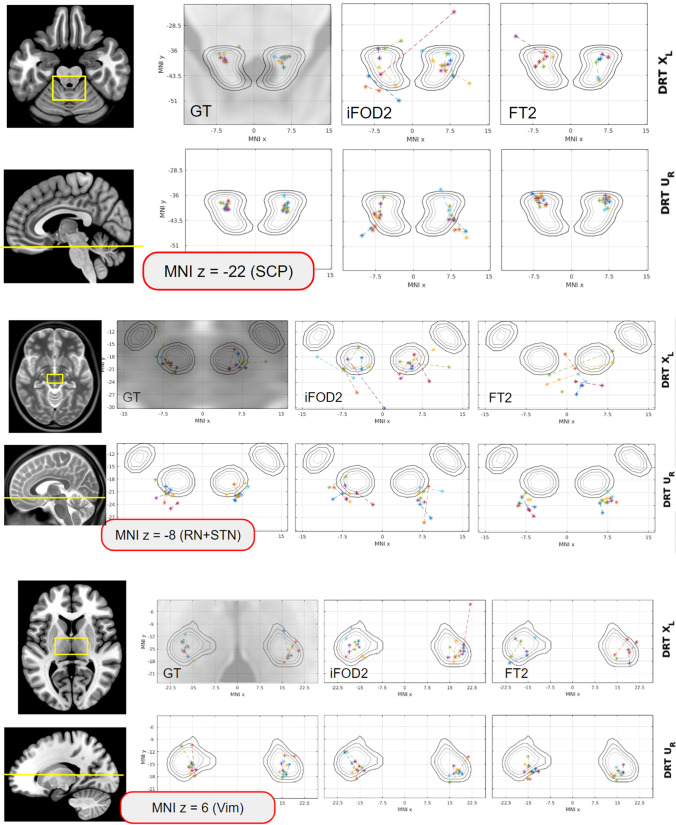
Anatomical validity. The center of gravity of the DRT is displayed at different z-levels in MNI space. Points connected by dotted lines indicate that the DRTs are from the same subject. Left to right: GT, iFOD2, FT2. Most variability is seen in the display of DRTx, the least variability is seen in DRTu which is displayed most reliably. MNI z =  − 22 representing the SCG level. For DRTu and FT2, the COGs are projected further anterior in SCG than for the other approaches. iFOD2 shows the most erratic COG projections of all approaches regardless of the display of DRTx or DRTu. MNI z =  − 8 representing the RN + STN level. At this level, the reconstruction of DRTx is the most erratic. For FT2, it can be seen that the DRT crossing itself is projected into this level, thus too far superficial. For DRTu, FT2 shows COG behind the RN NOT touching the nucleus. MNI z = 6 representing the Vim level. Again, most reliable penetration is shown for DRTu across all approaches. There is a tendency for DRTx to be projecting more to the center of the nucleus while DRTu appeared to project closer to its posterior border

To better understand the behavior of the different approaches at specific anatomical landmarks, where prior knowledge about the DRT course is available, we looked again at three z-levels but now with respect to characteristic anatomical structures (Fig. 4): (a) SCP, (b) Vim, and (c) RN/STN. In Fig. 7, scatter plots of the DRT COGs are shown for all different methods. For anatomical orientation, contours of nuclei templates are shown. The RN, STN, and Vim are derived from [23]; the superior cerebellar peduncle (SCP) was manually drawn.

## Discussion

In this contribution, we aim at the detailed evaluation of the single subject streamline rendition of the cerebello-thalamo-cortical tract/the DRT in a commercial stereotactic planning environment for the purpose of surgery planning. Outcome-parameters are anatomical accuracy, retest reliability, and comparison to other neuroscientific streamline rendition approaches.

### Dentato-rubro-thalamic tract anatomy

The DRT (Fig. 1) is the largest cerebellar output structure and in principle consists of two fiber pathways, namely the fasciculus cerebello-thalamicus (FCT) (or fasciculus thalamicus, fct) [28] and the postsynaptic cortical projection [43]. For the sake of simplification, we will here regard them as a single tract, the DRT. The DRT forms a connection of the dentate nucleus to the (contralateral) primary motor cortex (precentral gyrus) and also further anterior supplementary motor and prefrontal cortices. On its way to the cortex, the main DRT crosses over to the contralateral side thereby contributing to the decussation of Wernekinck [68]. A minimal part of the DRT continues ipsilateral to the precentral gyrus of the same side [10, 37, 41, 47]. The termination of the DRT is confined to the primary motor cortex (M1, PG) of the contralateral side [1–3, 14, 15, 17, 24]. There are authors who propose a thalamic sweet spot for DBS in essential tremor, which addresses fibers further anterior to supplementary motor regions [39]. According to some authors, distinct locations of the DRTu and DRTx are possible in the superior cerebellar peduncle, at the thalamic (DRTu more medial and posterior) and on the cortical level (DRTx more anterior and supplementary motor to prefrontal) [37, 47] making the discussion about individual anatomy display even more challenging.

Tractography-derived streamline renditions from dMRI represent sophisticated three-dimensional simulations of actual anatomy with an unclear level of anatomical accuracy. The anatomical tremor network has rich connections and includes brainstem and spinal projection pathways (amongst others) [43]. These rich connections are not visualized with current DTI technology on the single subject level. As a consequence, the tractographic rendition of the DRT represents only a part of this network [13–15]. There is emerging evidence that the DRT represents the very part of the tremor network which is accessible to therapeutic approaches like deep brain stimulation (DBS) and stereotactic lesion surgery (SLS), including MR-guided focused ultrasound (MRgFUS) [1, 3, 8, 12, 14, 15, 17, 21, 29, 32, 33, 39, 60, 64, 70]. Still, some of this work is retrospective in nature [1, 14, 39, 51, 55] and shows thalamic connectivity related to tremor improvement rather than the DRT as structure itself. It should be noted that the DRT has not yet reached the level of an established target for tremor surgery. Despite this fact, some groups have started to use the DRT in a prospective fashion for their targeting approaches [13, 17, 18, 20, 24, 53, 60].

The anatomical validity of the DRT rendition on the individual level is difficult to evaluate given the lack of a ground truth and variations in the reconstructions due to technical parameters (imaging sequences, imaging quality, field strength, movement artifacts). It is therefore of importance to understand the limitations of the technology [34, 53, 58, 63] used to create DRT streamline renditions which serve as a blueprint for surgical approaches [13, 18, 20, 24, 57]. The use of different imaging sequences, tracking algorithms [9], and other factors like unresolveable complex and intermingling fiber geometries have a great influence on the correctness of the depiction of a fiber tract with dMRI [11]. Moreover, besides a qualitative appreciation of the result of the streamline rendition, there is no method for directly checking the trueness of a fiber tract display on the single case basis. This might pose a problem on stereotactic planning because the quality and accuracy of the DRT’s rendition might have a direct influence on the success of the surgery. The consequences are even more important in lesioning approaches [8, 40] especially since lesions to the entire DRT or even parts of the structure have shown to cause detrimental clinical effects [8, 36].

Stereotactic and surgical planning systems typically use local tractographic approaches since these are fast and work with a minimal amount of manipulation [19, 44]. These local approaches and especially the deterministic tractography approach (DT), however, cannot simply resolve problems like kissing, branching, and crossing fibers [11]. A complex structure like the DRT synapses in the thalamic Vim (ventral intermediate nucleus)/VOP (ventralis oralis posterior nucleus) region onto a second neuron with a cortical termination in the motor cortex. Naturally, such synapses along with the (activation) direction cannot be displayed with the dMRI technology. A surgically and streamline-anatomically more important feature is the definition of the thalamic penetration level at the Vim nucleus with tractographic methods [1, 14, 57, 64] since the Vim nucleus and the adjacent subthalamic level are the classical target region [46] for any tremor reducing surgery [17, 21] especially in essential tremor (ET) and Parkinson’s disease (PD) tremor. These structures cannot directly be visualized in conventional anatomical sequences.

#### Dependence on chosen imaging sequence

We have assessed the impact of two MRI measurements of different quality (PRISMA vs. TRIO) on the rendition of the DRT under three tracking approaches. As exemplarily shown in Fig. 3, there is a clear qualitative dependence of DRT display. It is actually not surprising that the unguided approaches (iFOD2, GT) show some vulnerability to a lower quality sequence (TRIO). In this regard, the superior DRTu rendition (TRIO) under FT2 is most likely related to the re-iterative manual deletion of erroneous fibers (see the “Methods” section) and the introduction of prior anatomical knowledge, which was only performed for the FT2 approach.

#### Inter-method comparison

The approaches performed well with respect to DRTu. FT2 performed well at the SCP level while iFOD2 showed less congruence with this pattern. Best performance of all algorithms was seen in the RN + STN level (MNI z =  − 8). This is reassuring since for DBS treatment of ET typically a target just below the Vim in the STR is often chosen. At the level of the Vim, FT2 performed best but still showed a COG of 2 mm which needs to be taken into account during surgical planning.

#### Reproducibility

We have left out the thalamic nuclei Vim/VOI as potential waypoints for the individual tracking procedures (see the “Methods” section) and instead analyzed the results of DRT display with the approaches’ abilities to define these structures at the thalamic penetration level. In our analysis of reproducibility, we found that all approaches including the FT2 allowed for a reliable definition of DRTu especially at the here defined waypoints (SCP; RN + STN, Vim). These results point to a reliable DRTu display. A different result was found for DRTx: All approaches failed to show reliable reconstruction for the DRTx on a single case basis. According to this analysis, it cannot be recommended to display the DRTx on a single case basis for therapeutic (surgical) purposes.

#### Analysis of anatomical validity

For a check of anatomical correctness of the DRT rendition, certain anatomical waypoints (WP) may be taken into consideration like the dentate nucleus (DN, visible in T2W sequences), superior cerebellar peduncle (SCP, visible in T1W and T2W MRI sequences), red nucleus (visible in T2W MRI), and precentral gyrus (visible in T1W and T2W MRI sequences). For DRTx, the brachium conjunctivum (aka commissure of Wernekinck) [68] is visible in both T1W and T2W sequences although its true extent might be difficult to appreciate. We have here looked for a general tracing along these way points: For DRTu, the results were excellent for all analyzed approaches but best for the FT2 algorithm. It has to be noted that COGs of DRTu project anterior in SCG while preferentially more posterior in Vim nucleus. During implantation, this could potentially lead to a too far posterior placement of a DBS electrode or a lesion and should be further regarded. All approaches analyzed here showed erratic results for DRTx in the projection of COG (Fig. 7).

The analysis of DRT anatomy for their clinical validity and anatomical course with respect to atlas data has been performed before [45] who focused in their approach to the problem on the choice of ROIs/VOIs. The authors showed similar results for streamline display of the DRT and were equally unsuccessful for the detection of DRTx. They used a deterministic (DT) and a probabilistic algorithm (PT) for DRT streamline rendition and showed rather volatile results with respect to the individual patient but with a congruence between the main structures and the distinct algorithms in native space (analysis in MNI space was not performed). PT was better able to show DRTx. Their contribution did not reliably show a congruence between DRT rendition and tremor outcome [45]. On the contrary, Fenoy et al. have successfully used deterministic tracking prospectively in their approach (using Medtronic Stealth Viz DTI) to successfully guide tremor DBS in ET [25]. These results are underpinned by our own and previous results of DRT DBS for tremor in various indications in the same software environment (StealthViz DTI) [13–15]. With the application of a more complicated statistical model, a correlation between tremor reduction and DRT to contact proximity was found [14, 17]. So in principle, an effect of a different software and tractographic algorithms is thinkable [9]. On the contrary, we have ourselves successfully used a similar software environment (Elements) for DRT rendition under the difficult conditions of revision surgery [20] and have successfully done so also for another target structure [19].

Lastly, an anatomically correct DRT display would warrant a correct rendition of the crossed pathway [10, 37, 43] on the single subject level. However, studies which looked at tremor improvement and targeted DRT have almost exclusively used DRTu with good results [13, 17, 20, 24, 33, 57, 60].

### Limitations

**Fig. 8:**
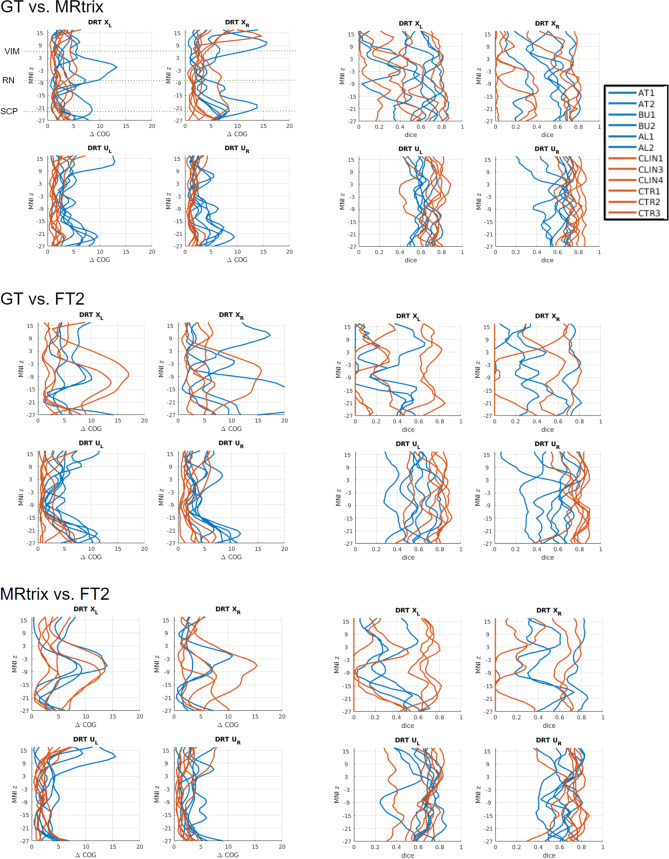
Detailed inter-method comparison: for all three method pairings, COG differences and dice coefficients were computed along the MNI z-coordinate. Results from the 6 TRIO datasets are indicated by blue lines; results from the 6 PRISMA subjects are shown in orange. In general, agreements for the uDRT are better than xDRT. With respect to uDRT the GT vs. MRtrix agree best, while MRtrix vs. FT2 shows the most disagreement. Results from GT form a good tradeoff between MRtrix and FT2

**Fig. 9:**
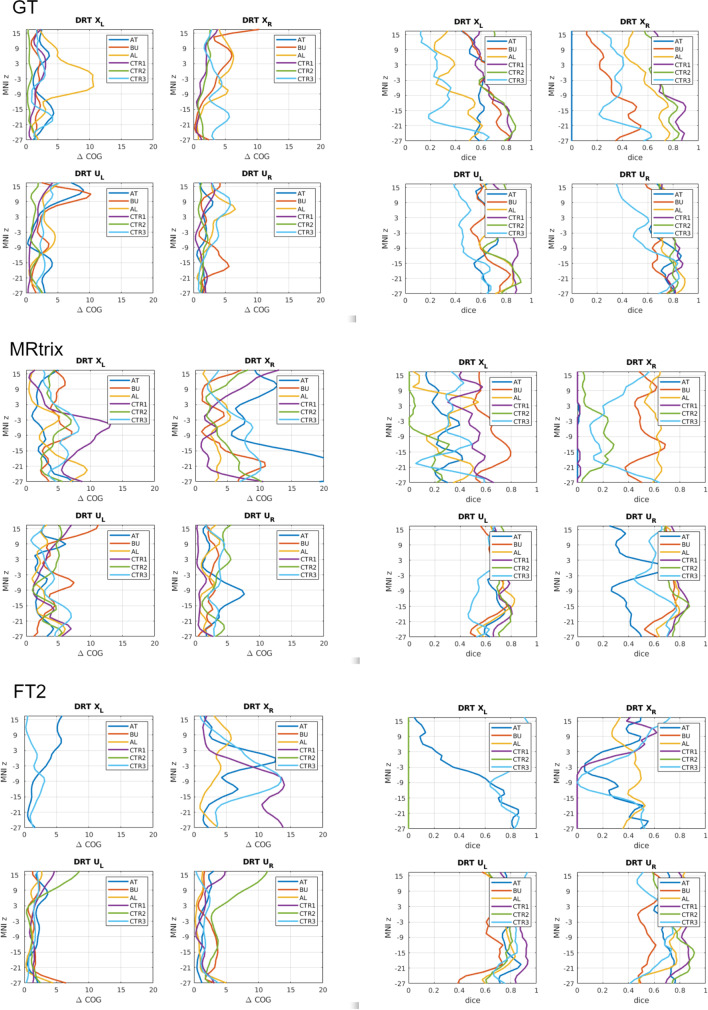
Detailed reproducibility analysis showing COG differences and dice coefficients between two scans from the same individual. Three subjects from TRIO (AT, BU, AL) and three subjects from PRISMA (CTR1, CTR2, CTR3) are shown in different colors. For the uncrossed DRT, the manual FT2 approach outperforms GT and MRtrix with very small deviations (< 2.5 mm) at most levels. On the other hand, the crossed DRT is hardly reconstructed by FT2

There appear to be unresolved anatomical issues with respect to the prospective definition of the DRT as a target structure for tremor surgery: The termination of the DRT (crossed and uncrossed) in the precentral gyrus (M1) is at this moment not accepted by all groups [38, 39] and a matter of debate [2]. However, since most studies lean towards a projection of tremor-improvement-related fibers to M1 [1, 3, 32, 51], and since anatomy is rather decisive [28, 43, 56], we chose to stick to our original approach [15]. We would like to point to the fact that there might be a difference for the course of DRTx/DRTu fibers with respect to the anatomical waypoints (SCP, Vim). It is in our eyes not fully clear if this is a bias inherent to the distinct fiber tracking approach or if this reflects distinct anatomy [37, 47]. The latter case would have a dramatic influence on the targeting strategy chosen, and we have ourselves earlier proposed to only target the anterior half of the DRT at the thalamic and adjacent subthalamic levels [14]. There was no clinical data included in this study which could prove the effectiveness of DRT rendition and DRT stimulation. This was, however, not the aim of this study which solely looked at the streamline rendition of the DRT in different environments and under retest conditions. The number of subjects/images considered here is low, which challenges the generalizability of the results. However, when looking at the individual data (in Figs. 8 and 9), the trends are obvious and reasonable. Lastly, the FT2 method was the only one which was assessed allowing refinement steps in cutting erroneous fibers. However, since the unrefined FT2 resulted in a plethora of fibers, a comparison with other methods was not feasible.

## Conclusion

We have here scrutinized the streamline depiction of a clinically researched target for tremor surgery—the DRT—in different tractography environments using dMRI in 9 control subjects. In general, the uncrossed DRT (DRTu) can be depicted with good quality. FT2 (surgical) and GT (neuroscientific) show high congruence. It therefore appears to be justified to use the FT2 approach for the surgical planning on the single subject level when targeting DRTu. While GT shows acceptable results for DRTx, the crossed pathway cannot reliably be reconstructed with the other (iFOD2 and FT2) algorithms. Especially for FT2, a reconstruction of DRTx shows the crossing too far superficial, which points to an inclusion and jumping to different fiber tracts. FT2 is useful especially because of its manual editing possibilities of cutting erroneous fibers on the single subject level. An uncertainty of 2 mm as mean displacement of DRTu COG is to be expected and should be respected when using this approach for surgical planning. Especially, the evaluation of waypoints appears to be helpful for appreciation of anatomical validity.

## Abbreviations

AC: Anterior commissure
AL: Ansa lenticularis
B0: Diffusion weighting strength factor (B0 means no weighting)
BS: Brain stem
COG: Center of gravity
CSD: Constrained spherical deconvolution
CTT: Cerebello-thalamic tract
CTR: Control
cZI: Caudal zona incerta
DBS: Deep brain stimulation
DICOM: Digital Imaging and Communications in Medicine (imaging standard)
DN: Dentate nucleus
DRT(T): Dentato-rubro-thalamic tract (FCT + thalamocortical projection)
DRTu: Uncrossed DRT(T)
DRTx: Crossed DRT(T)
DT: Deterministic tracking
DTI: Diffusion tensor magnetic resonance imaging
dMRI: Diffusion-weighted magnetic resonance imaging
EPI: Echo-planar imaging
ET: Essential tremor
FA: Fractional anisotropy
FACT: Fiber assignment for continuous tracking
FCT: Fasciculus cerebellothalamicus (aka fasciculus thalamicus)
FT2: 2 Tensor deflection algorithm (deterministic)
GT: Global tracking
iFOD2: Probabilistic tracking based on constrained spherical deconvolution (CSD)
ML: Medial lemniscus
MNI: Montreal Neurological Institute
MRgFUS: Magnetic resonance imaging-guided focused ultrasound
MRI: Agnetic resonance imaging
ot: Of thalamus
PC: Posterior commissure
PCA: Principal component analysis
PD: Parkinson’s disease
PFC: Prefrontal cortex
PG: Precentral gyrus
PMC: Premotor cortex
PoG: Postcentral gyrus
pSTR: Posterior subthalamic region
PT: Probabilistic fiber tracking
RN: Red nucleus
SCP: Superior cerebellar peduncle
SLS: Stereotactic lesion surgery
SNR: Signal to noise ratio
STN: Subthalamic nucleus
STR: Subthalamic region targets
VCA: Ventral caudal anterior nucleus (of thalamus)
VCP: Ventral caudal posterior nucleus (of thalamus)
Vim: Ventral intermediate nucleus (of thalamus)
VOA: \Ventral oral anterior nucleus (of thalamus)
VOI: Volume of interest
VOP: Ventral oral posterior nucleus (of thalamus)
WM: White matter
